# Effects of Qingda granule on patients with grade 1 hypertension at low-medium risk: study protocol for a randomized, controlled, double-blind clinical trial

**DOI:** 10.1186/s13063-022-07006-0

**Published:** 2023-01-02

**Authors:** Lin-zi Long, Jian-feng Chu, Hua Qu, Qiao-ning Yang, Yan Lu, Chang-geng Fu, Jun Peng, Ke-ji Chen

**Affiliations:** 1 Academy of Integrative Medicine, Fujian University of Traditional Chinese Medicine, Fuzhou, 350112 China; 2 Department of Geriatrics, Xiyuan Hospital, China Academy of Chinese Medical Sciences, Beijing, 100091 China; 3 Chen Keji Academic Thought Inheritance Studio, Fujian University of Traditional Chinese Medicine, Fu Zhou, China; 4 Fujian Key Laboratory of Integrative Medicine on Geriatrics, Fujian University of Traditional Chinese Medicine, Fu Zhou, China; 5 National Clinical Research Center for Chinese Medicine Cardiology, Xiyuan Hospital, China Academy of Chinese Medical Sciences, Beijing, China; 6 Department of Cardiovascular Disease Center, Xiyuan Hospital, China Academy of Chinese Medical Sciences, Beijing, 100091 China

**Keywords:** Hypertension, Qingda granule, Blood pressure, Randomized controlled trials

## Abstract

**Background:**

Numerous pre-clinical studies showed that Qingda granule (QDG) was effective in treating hypertension. This study aims to evaluate the efficacy and safety of QDG in reducing blood pressure among patients with grade 1 hypertension at low-medium risk.

**Methods:**

The study is designed as a randomized, multi-center, double-blinded, non-inferiority clinical trial. Five hundred fifty-two patients with grade 1 hypertension at low-medium risk from 13 hospitals will be recruited and randomly assigned to the QDG group (*n* = 276, treated with valsartan capsule simulation agent and QDG) or control group (*n* = 276, treated with valsartan capsule and QDG simulation agent). The treatment period will be 4 weeks and the follow-up period will last 4 weeks after treatment. Primary outcome will be a decreased value of systolic blood pressure and diastolic blood pressure after treatment. And second outcome will include the decreased value of diastolic blood pressure and systolic blood pressure at the end of follow-up, the percentage of participants achieving normal blood pressure at the end of treatment and follow-up, the Hamilton Anxiety Scale and TCM syndrome scores at the end of treatment and follow-up, and levels of hypertensive hormones at end of treatment and follow-up.

**Discussion:**

This study will provide initial evidence regarding the clinical efficacy and safety of QDG in treating grade 1 hypertension at low-medium risk.

**Trial registration:**

Chinese Clinical Trial Registry ChiCTR2000033890. Registered on 15 June 2020.

**Supplementary Information:**

The online version contains supplementary material available at 10.1186/s13063-022-07006-0.

## Background

About 1.13 billion individuals suffer from hypertension around the world, with fewer than 20% of them being controlled to normal blood pressure [1]. Previous studies demonstrated that hypertension could increase the risk of coronary heart disease, stroke, and end-stage renal disease [2–4]. Although antihypertensive drugs are effective in treating hypertension and decreasing hypertension-associated risks, the side effects of these drugs often result in the discontinuation of the prescription [5, 6].

Traditional Chinese medicine (TCM) has shown efficacy in treating hypertension without severe side effects [7]. In particular, *Qingxuan Jiangya* decoction (QXJYD) has been used for the treatment of hypertension for many years in clinical practice [8]. QXJYD can decrease blood pressure and arterial vascular remodeling in spontaneously hypertensive rats [8, 9].

*Qingda* granule (QDG) formulation, simplified from QXJYD. The main drug components include *Gastrodia elata*, *Uncaria*, *Scutellaria baicalensis*, and lotus germ, which were shown to attenuate elevated blood pressure and promote vasorelaxation of thoracic aortic rings in the animal study with spontaneously hypertensive rats [10]. Some QDG components such as rhynchophylline, gastrodin, and baicalin have antihypertensive effects [9, 11, 12]. These findings have provided important evidence to further validate the anti-hypertensive effects of QDG in clinical trials. In the currently multi-center, double-blinded, randomized controlled non-inferiority trial, the objective is to evaluate the effects and safety of QDG compared with valsartan.

## Design and method

### Study setting

**Fig. 1:**
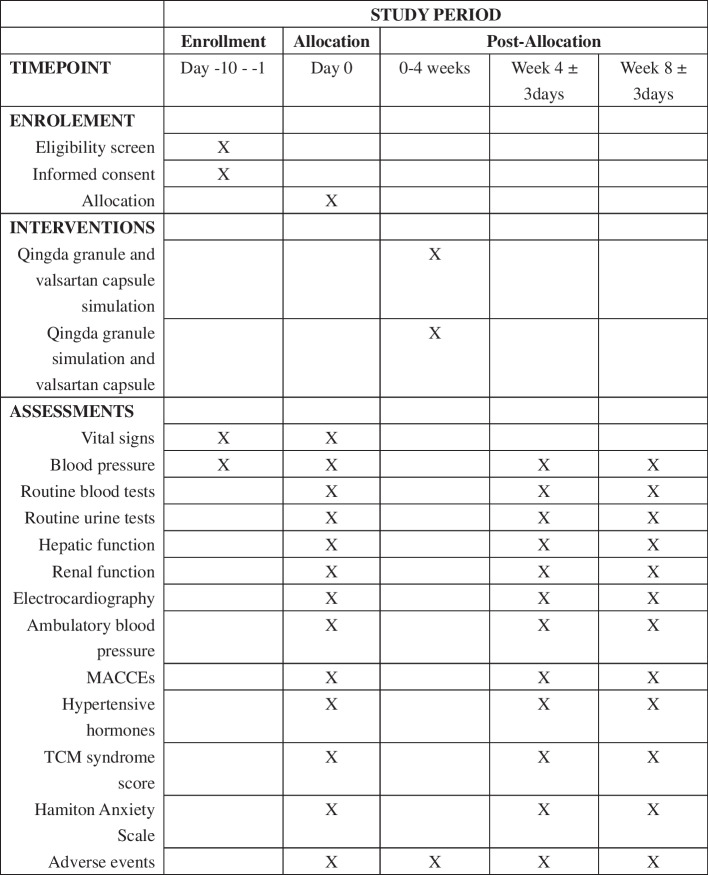
SPIRIT schedule of enrolment, interventions, and assessments. Abbreviations: MACCEs, major adverse cardiac and cerebrovascular events; TCM, traditional Chinese medicine

**Fig. 2:**
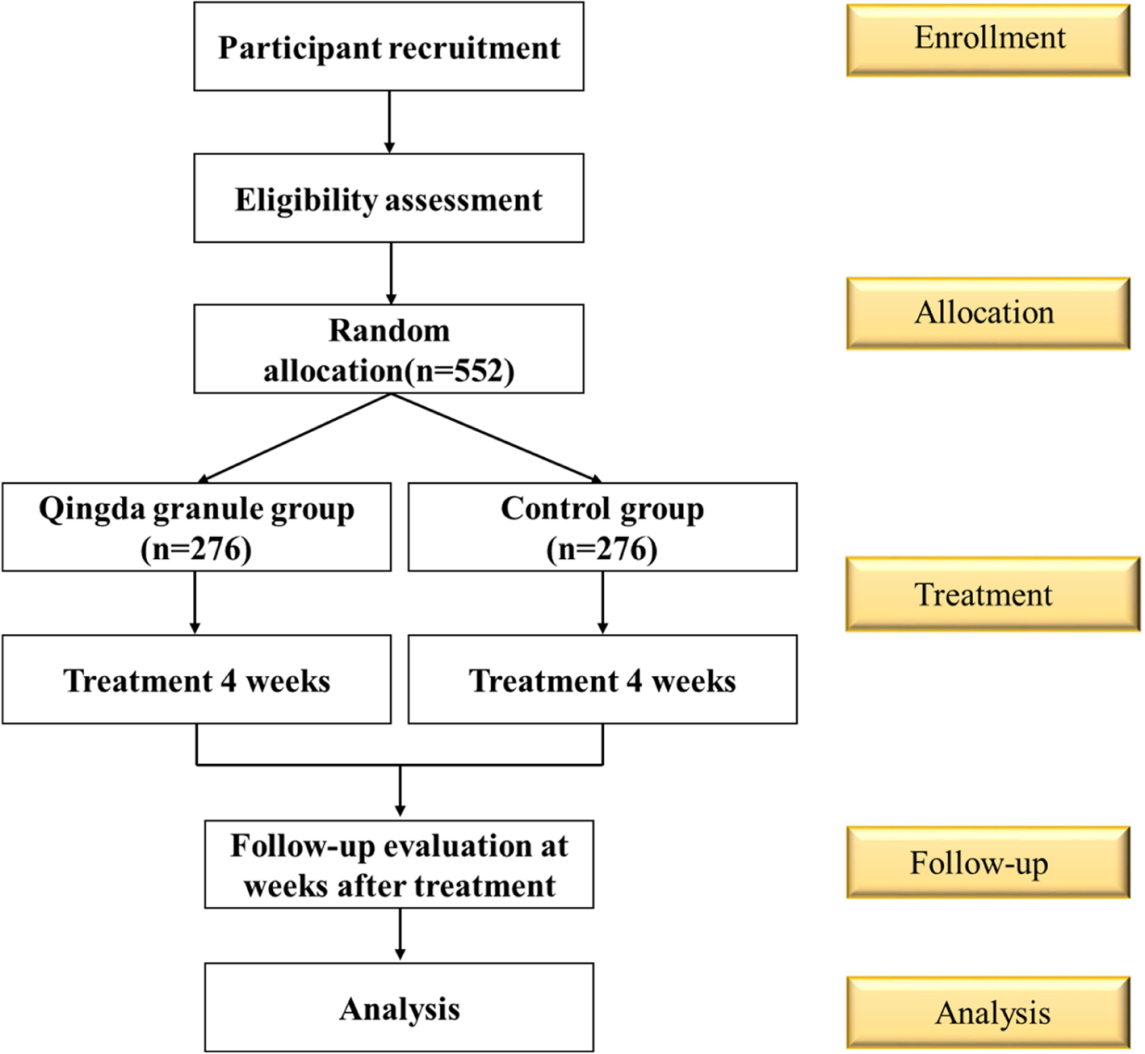
Study flow chart

The study is a randomized, multi-center, double-blinded, non-inferiority clinical trial. The protocol for the trial was approved by the Medical Ethics Committee of Xiyuan Hospital of the Chinese Academy of Chinese Medical Sciences (2020XLA027-3) and registered in the Chinese Clinical Trial Registry (http://www.chictr.org.cn/showproj.aspx?proj=55279) with number ChiCTR2000033890 on 15 June 2020. The protocol is written in accordance with the Standard Protocol Items: Recommendations for Interventional Trials (SPIRIT), showed as Supplementary File 1 [13]. And the schedule of enrolment, interventions, and assessments is shown in Fig. 1 and the study flowchart is shown in Fig. 2.

### Participants

Inpatients and outpatients diagnosed with grade 1 hypertension (at low-medium risk) from 13 hospitals (Supplementary File 2) will be screened based on the inclusion and exclusion criteria. The researchers will recruit inpatients and outpatients and gain the informed consent from the patients during the admission to the outpatients or to the hospital.

### Inclusion criteria

1. Grade 1 hypertension (low-medium risk) is diagnosed according to the Chinese Guidelines for the Prevention and Treatment of Hypertension [14].
2. The participants do not take any antihypertensive medicine within 1 month of starting the trial.
3. The age of the participants ranges from 20 to 75 years (inclusive).
4. Participants are informed about the trial and voluntarily sign a consent form.

### Exclusion criteria

1. Participants with obvious liver or kidney diseases, alanine transaminase (normal value: 0–40U/L) and aspartate transaminase values (normal value: 29–35U/L) 1.5 times higher than the upper reference limit, or blood creatinine and urea nitrogen levels higher than the upper reference limit.
2. Participants with serious mental illness, hematopoietic disease, malignant tumor, or other major diseases.
3. Women currently pregnant or preparing for pregnancy.
4. Involvement in other clinical trials within 3 months before enrollment.
5. Suspected or definite allergy to the ingredients of the study drug.

### Withdrawal, dropout, and discontinuation

Enrolled participants that fail to complete all the assessments of the trial, regardless of when or why, will be considered to have withdrawn. Reasons for withdrawal will be recorded on case report forms (CRFs), and data for these participants will be included in the final analysis only up to the last follow-up.

The trial can be terminated in the following cases: (1) participant(s) develop grade II or III hypertension, and other antihypertensive medicines should be added based on treatment guidelines [15], (2) no effects of the test drugs are observed during the trial, or a decision to terminate is taken (3) based on financial or management reasons or (4) by administrative authorities.

### Sample size

The target sample size was calculated according to the decreased value of systolic blood pressure (12.9±11.8 mm Hg) observed after 8-week treatment with valsartan capsules in a randomized clinical trial [16], and the non-inferiority margin of QDG has been established as −3 mm Hg. The decreased value of QDG on systolic blood pressure was 12.0±10.5 mm Hg, which was calculated based on our clinical practice. The minimal sample size to achieve a type I error rate of *α* = 0.05 and a power of 80% (type II error rate *β* = 0.2) is 440 participants. The size was calculated using the formula:

$$n=\frac{{\left({Z}_{1-\alpha }+{Z}_{1-\beta}\right)}^2\times \left({\upsigma}_1^2+{\upsigma}_2^2\right)}{{\left(\varepsilon -\delta \right)}^2}$$

where σ_1_ and σ_2_ are the standard deviations of the QDG and control groups, respectively; *ε* is the mean difference between the two groups; δ is the non-inferiority margin; *α* is the type I error rate; and *β* is the type II error rate. The study will strive to enroll 550 participants to compensate for a dropout rate as high as 20%. For convenience of randomization, we will aim to recruit 552 patients.

### Recruitment

Participants will be recruited in 13 hospitals (Supplementary File 2). Patients willing to participate in the trial will be screened for eligibility by a clinical researcher. Eligible patients will receive detailed information about the clinical trial. Patients providing consent to the clinical study terms will participate in the trial and will be randomly assigned to the QDG or control group.

### Randomization and blinding

Participants will be randomized in a 1:1 ratio to the QDG or control group using a computer-generated, site-stratified, block randomization schedule. The study drugs will be labeled with sequential randomization numbers, and the participant will be assigned the lowest number available at each hospital. All participants, care providers, and attending physicians will be blinded to the assignment until the trial is finished. The trial-group assignment will be concealed from all participants, clinicians, and investigators throughout the trial. To ensure blinding, randomization sequences will be generated by statisticians and then kept in identical, opaque, sealed, sequentially numbered envelopes. Only the trial’s pharmacist has access to the randomization list.

### Intervention

The course of treatment will last 4 weeks, and patients will be followed up for 4 additional weeks to assess any persistent effects of the drug. Participants will be examined at week 0 (baseline, visit 2), week 4 (end of treatment, visit 3), and week 8 (end of follow-up, visit 4). The QDG group will be treated with valsartan capsule simulation agent (80 mg, once daily for 4 weeks) and QDG (5 g, twice daily for 4 weeks), while the control group will be treated with valsartan capsule simulation agent (80 mg, once daily for 4 weeks) and QDG simulation agent (5 g, twice daily for 4 weeks). The drugs and simulation agents will be provided by Jiangyin Tianjiang Pharmaceutical (Jiang’yin City, China). The simulation agent will be prepared using soluble starch, colorant, and a bitter additive to achieve appearance, smell, and taste comparable to the drug. The drugs and simulation agents will be identical in outer packaging, color, shape, and flavor. The drug that patients did not take would be returned to the researcher. And no other anti-hypertension drugs should be taken during the study process.

### Outcomes assessment

#### Primary outcome

After 4 weeks’ treatment, the decreased values of clinic systolic blood pressure and diastolic blood pressure after treatment are used as primary outcome (the difference between visits 3 and 2). Clinic blood pressure was measured using an automated sphygmomanometer (Omron, HEM-7121) in the right arm with an appropriate cuff size. The measurements were taken after the participants rested in a sitting position for 5 min, at least 1 h after the subject’s last meal, and at least 30 min after smoking or consuming caffeinated beverages. To obtain accurate data, the measurements will be conducted 3 times, and the mean value will be used as the outcome.

#### Secondary outcomes

1. The decreased value of clinic systolic and diastolic blood pressure after 4-week follow-up (the difference between visits 4 and 2).
2. The percentage of participants achieving normal clinic blood pressure (systolic blood pressure ≤120 mm Hg and diastolic blood pressure ≤80 mm Hg) by the end of treatment (visit 3) and at the end of follow-up (visit 4).
3. The mean systolic/diastolic blood pressure during 24-h, daytime, and nighttime at the end of treatment (visit 3) and end of follow-up (visit 4).
4. The Hamilton Anxiety Scale and TCM syndrome scores at the end of treatment (visit 3) and end of follow-up (visit 4).
5. Levels of hypertensive hormones (renin, aldosterone, cortisol, and corticotropin) at the end of treatment (visit 3) and end of follow-up (visit 4).

Clinic blood pressure will be measured as described above. Ambulatory blood pressure monitoring was performed using validated oscillometric monitors (Meditech, ABPM-05). And hypertension hormone (renin, aldosterone, cortisol, and corticotropin) will be collected in the morning fasting after lying down for 1-h venous blood, using the immunoassay.

### Safety

At visits 2–4, safety data will be collected based on vital signs (respiratory, heart rate, and body temperature) and electrocardiography (ECG). We will request consent for the collection of blood and urine samples to assess hepatic and renal function. The blood and urine samples will be collected at 13 hospitals and stored at room temperature. After completion of sample collection, laboratory analysis will be conducted in the laboratories of hospital. The remaining samples will be destroyed after being examined. Adverse events (AEs), defined as negative or unintended clinical manifestations following treatment, will be observed and recorded throughout the trial. If an AE occurs, the clinical researcher will adopt appropriate measures to guarantee the safety of participants. If a serious AE occurs, the clinical researcher will report it to the Medical Ethics Committee and a decision will be taken whether to terminate the trial. When the patients develop to grade II or III hypertension, adverse events (AEs) will be reported to the data monitoring committee as well as the medical ethics committee. The severity and causality will be estimated by researchers.

### Data management

The researcher will collect information on participants enrolled in this study and the data will be input into a validated database which identified participants by a participant code rather than participant names. Database lock will occur once quality assurance procedures have been completed. Once data are input into the database, data validation checks will be performed via computer. The changes to the study data will be recorded. Any data required to support the protocol can be supplied on request.

The database is safeguarded against unauthorized access by established security procedures and supported by the database administrator in conjunction with any updates or changes to the database. Data for analysis is locked and cleaned according to established procedures.

A data monitoring committee will be formed, which include the corresponding author, 1 independent biostatistician, and 2 independent expert members specialized in clinical trials. The goal of the committee is to regularly review and maintain the confidentiality of the accumulating data. In addition, the committee is also formed to safeguard the interests of the participants. The committee will meet once every 3 months over the course of the trial to check the data of the trial.

### Interim analysis

An interim analysis will be performed for the primary outcome and adverse events after starting the trial for 1 year. During the interim analysis, the trial will be continued. The trial will be terminated early if clear benefit or harm of the treatment is shown after the interim analysis.

### Statistical analysis

The data analysis will be based on the principle of intention to treat (ITT), and potential missing values will be filled in using data from the last observation. Data will be presented as mean ± standard deviation, median with range, or number and percentage. Data will be analyzed using SAS 9.3 software (SAS Institute, Cary, NC, USA) using two-sided tests and a significance level of *p* < 0.025. For comparison between groups, two independent sample *T*-test (group *T*-test ) and Wilcoxon rank sum test were used for measurement data. The Pearson chi-square test or Fisher exact test was used for counting data. Wilcoxon rank sum tests were used for grade data. For intra-group comparison, paired *T*-tests or Wilcoxon symbolic rank test was used for measurement data. CMH chi-square tests were used when considering the central effect. Sub-group analyses will be conducted based on the characteristics of included participants.

### Protocol amendments

Protocol will be amended according to the terms of the medical ethical committee application if necessary. Xiyuan Hospital, China Academy of Chinese Medical Sciences, is the competent authority. The protocol amendments will be approved by the competent authority and recorded in other centers. In some conditions, we will perform protocol amendments to ensure the safety of the subjects of the trial and the quality or safety of any intervention used in the trial. All amendments will be notified to the medical ethical committee application and to the competent authority. Any deviations from the protocol will be fully documented using a breach report form.

## Discussion

QXJYT was created by Academician Chen Keji. After years of clinical practice, it has been proved to have a certain antihypertensive effect, which can significantly improve the clinical symptoms and quality of life of hypertension. At the same time, it has an obvious protective effect on target organs [17]. QDG is composed of *Gastrodia elata*, *Uncaria*, *Scutellaria baicalensis*, and *lotus seed heart* according to more than 60 years’ clinical experience of Academician Chen Keji in addition and subtraction of QXJYT [18]. In the present clinical trial, the effects of QDG on grade 1 hypertension (low-medium risk) will be investigated. QDG, derived from QXJYD, showed obvious effects on decreasing blood pressure and promoting the vasorelaxation of thoracic aortic rings in spontaneously hypertensive rats [10]. Further mechanistic investigation demonstrated that QDG ameliorated Ang II-induced hypertension in mice and inhibited proliferation of vascular smooth muscle cell of aorta via suppressing the activation of MAPKs and PI3K/AKT pathways [19]. In addition, some components of QDG, such as rhynchophylline, gastrodin, and baicalin, also have the effects of antihypertension [9, 11, 12]. These results serve as a solid foundation for the present clinical trial investigating the effects of QDG on grade 1 hypertension with low-medium risk. The present clinical study is designed as a randomized, multi-center, double-blinded, placebo-controlled, non-inferiority trial, which will provide high-quality evidence for the further clinical practice of QDG.

The clinical trial will be performed in China and will enroll Chinese patients, and the 13 hospitals will mainly serve Han Chinese, so it will not evaluate the effects of QDG in other ethnic groups. The follow-up period is relatively short, and therefore, some effects may be potentially missed.

## Trial status

The trial is currently in the status of data analysis. The study started recruiting participants in May 2020 and ending in December 2021. The protocol version number is ChiCTR2000033890 and the date is June 15, 2020. The protocol has been submitted in October 2020; unfortunately, the manuscript was submitted as a research article but it has since been identified as a Study Protocol article. The journal found the question after 2 years and required us to re-submit the manuscript in September 2022. So the manuscript was published late.

## Supplementary Information

**Additional file 1.** SPIRIT Checklist.

**Additional file 2.** Attended hospitals.

## Data Availability

The datasets analyzed during the current study and statistical code are available from the corresponding author on reasonable request, as is the full protocol.
