# Supplementary material for: Effects of Qingda granule on patients with grade 1 hypertension at low-medium risk: study protocol for a randomized, controlled, double-blind clinical trial
Source: Trials. 2023 Jan 2;24:1. doi: 10.1186/s13063-022-07006-0 (PMC9806902; doi:10.1186/s13063-022-07006-0)
Supplement: Supplementary file 2 — Additional file 2. Attended hospitals. [file 13063_2022_7006_MOESM2_ESM.doc]

**Attended Hospital**

| **Number** | **Name** |
| --- | --- |
| 1 | Xiyuan Hospital, China Academy of Chinese Medical Sciences |
| 2 | Dongzhimen Hospital, Beijing University of Traditional Chinese Medicine |
| 3 | Guang 'anmen Hospital, China Academy of Chinese Medical Sciences |
| 4 | Beijing Anzhen Hospital, Capital Medical University |
| 5 | Traditional Chinese Medicine Hospital of Guangdong Province |
| 6 | Xiamen Hospital of Traditional Chinese Medicine |
| 7 | Fujian People's Hospital |
| 8 | Shandong Provincial Hospital of Traditional Chinese Medicine |
| 9 | Wuxi Traditional Chinese Medicine Hospital |
| 10 | Beijing Integrated Traditional Chinese and Western Medicine Hospital |
| 11 | Ruikang Hospital Affiliated to Guangxi University of Chinese Medicine |
| 12 | The First Affiliated Hospital of Henan University of Chinese Medicine |
| 13 | Nantong Hospital of Traditional Chinese Medicine |
